# Immune crosstalk in Alzheimer’s and Parkinson’s disease: insights from Drosophila models into the brain–peripheral immune axis

**DOI:** 10.3389/fimmu.2026.1725046

**Published:** 2026-01-27

**Authors:** Faiza Parvez, Rahul

**Affiliations:** Neurobiology Lab, Department of Zoology, Science Faculty, University of Allahabad, Prayagraj, India

**Keywords:** Alzheimer’s disease, *Drosophila melanogaster*, immune crosstalk, innate immunity, neurodegeneration, neuroinflammation, Parkinson’s disease, toll pathway

## Abstract

**Background:**

Neurodegenerative diseases (NDs) such Alzheimer’s disease (AD) and Parkinson’s disease (PD) are increasingly understood as systemic disorders driven by chronic neuroimmune dysregulation. The bidirectional communication between the central nervous system (CNS) and peripheral immune compartments is termed neuroimmune crosstalk, plays a pivotal role in disease initiation, progression, and therapeutic resistance. However, mammalian models often obscure mechanistic resolution due to immune redundancy and adaptive complexity.

**Objective:**

This review highlights *Drosophila melanogaster* as a genetically tractable and evolutionarily conserved model for dissecting innate immune signaling and inter-organ communication in neurodegeneration. We emphasize its utility in resolving causality, identifying conserved cytokine pathways, and modeling systemic inflammation relevant to Parkinson’s and Alzheimer’s disease.

**Key findings:**

*Drosophila* possesses a tripartite immune system that is brain-resident glia, circulating hemocytes, and the fat body that coordinates responses via Toll, Immune deficiency (Imd), JAK/STAT, and MAPK pathways. Glial cells engage in Draper-mediated phagocytosis and NF-κB/Relish signaling, while peripheral immune components modulate CNS integrity through cytokines such as Unpaired 3 (Upd3) and Eiger. Furthermore, hyperactivation of the Imd pathway’s NF-κB homolog, Relish, within the CNS drives neurodegeneration via the neurotoxic effects of Antimicrobial Peptides (AMPs). These mechanisms mirror mammalian neuroimmune dynamics and reveal conserved therapeutic targets.

**Conclusion:**

*Drosophila melanogaster* offers unparalleled mechanistic clarity in modeling neuroimmune interactions. Its simplified immune architecture, precision genetics, and compatibility with multi-omics and AI-assisted phenotyping position it as a strategic complement to vertebrate models. Insights from *Drosophila* are redefining neurodegeneration as a multi-organ process and accelerating the development of inflammation-targeted therapies for ND.

## 1 Introduction

Neuroimmune crosstalk refers to the dynamic, bidirectional communication between the central nervous system (CNS) and the immune system, encompassing molecular signals, cellular interactions, and systemic feedback loops. Under physiological conditions, this interplay is essential for maintaining neural homeostasis. It regulates synaptic pruning, facilitates the clearance of apoptotic debris, and supports neurovascular integrity through tightly controlled glial–immune coordination (1). However, in neurodegenerative disorders such as Alzheimer’s disease (AD) and Parkinson’s disease (PD), this communication becomes chronically dysregulated. The result is a persistent, non-resolving inflammatory state often termed **“**inflammaging**”** which exacerbates neuronal vulnerability and accelerates disease progression. This sterile inflammation is driven by sustained activation of microglia and astrocytes, which shift from neuroprotective to neurotoxic phenotypes, releasing pro-inflammatory cytokines (e.g., IL-1β, TNF-α, IL-6), reactive oxygen species, and matrix-degrading enzymes (2, 3).

Importantly, neuroimmune crosstalk is not confined to the CNS. Peripheral immune signals originating from the gut, spleen, or systemic circulation can influence brain function via cytokine diffusion, blood–brain barrier modulation, and immune cell infiltration. Conversely, CNS-derived stress signals can alter peripheral immune tone, creating a feedback loop that reinforces neuroinflammation and systemic immune imbalance (1, 2). This bidirectional axis is increasingly recognized as a mechanistic bridge linking hallmark neurodegenerative features such as protein aggregation (Amyloid-β, Tau, α-synuclein), mitochondrial dysfunction, and oxidative stress with immune dysregulation. Understanding the molecular mediators of this crosstalk, including extracellular vesicles, chemokines, and glial–immune interfaces, is crucial for identifying therapeutic targets that can modulate inflammation without compromising immune defense. Emerging strategies such as microglial replacement, JAK/STAT modulation, and cytokine-targeted interventions offer promising avenues for disease modification (3, 4).

Rodent models have long served as the cornerstone of neurodegenerative disease research, offering valuable insights into protein aggregation, synaptic dysfunction, and behavioral phenotypes. However, these models often fall short when dissecting the mechanistic underpinnings of innate immune signaling. For example, many transgenic mouse models of AD are built around the Amyloid Cascade Hypothesis, which oversimplifies disease etiology and fails to capture the full spectrum of multifactorial pathology observed in human AD and PD, including systemic inflammation, mitochondrial dysfunction, and glial heterogeneity (5, 6). Moreover, the complexity of the mammalian immune system with its diverse glial subtypes, adaptive immune components, and redundant signaling networks can obscure the causal effects of manipulating individual innate pathways. This redundancy often limits the interpretability of targeted interventions, especially when attempting to isolate the contributions of conserved modules such as NF-κB or JAK/STAT (7).

In contrast, *Drosophila* offers a reductionist yet evolutionarily conserved platform for neuroimmune research. The fly possesses only innate immunity, yet retains core signaling pathways including Toll, Immune deficiency (Imd), and JAK/STAT that are homologous to mammalian counterparts (8, 9). This streamlined architecture enables precise dissection of inflammatory cascades without the confounding influence of adaptive immunity. Importantly, *Drosophila* provides robust genetic tools for tissue-specific and temporally controlled manipulation of immune and neurodegenerative pathways. Techniques such as GAL4/UAS, RNAi, CRISPR/Cas9, and temperature-sensitive alleles allow researchers to model proteinopathies (e.g., Tau, α-synuclein) and evaluate their impact on glial activation, cytokine signaling, and blood–brain barrier integrity (3, 10). This versatility positions *Drosophila* as a strategic complement to vertebrate models, particularly for inflammation-led drug discovery. Its short lifespan, low cost, and high-throughput screening capacity make it ideal for identifying modulators of neuroimmune interactions and translating molecular insights into therapeutic leads (11).

The immune architecture of *D. melanogaster* is compartmentalized into three interlinked systems: brain-resident glial cells, circulating hemocytes, and the fat body. These components engage in continuous molecular dialogue to maintain organismal homeostasis and coordinate responses to neurodegenerative stress (3, 8, 12). Glial cells, residing within the CNS, serve as functional analogs to mammalian microglia and astrocytes. They regulate neurotransmitter clearance, maintain the blood–brain barrier, and respond to neural injury through cytokine release and phagocytic activity (12). Upon encountering neurodegenerative cues such as misfolded proteins or oxidative stress, glia initiates local immune responses and signal to peripheral compartments. Hemocytes, the fly’s circulating immune cells, are developmentally and functionally analogous to vertebrate macrophages and myeloid cells. They patrol the hemolymph, engage in phagocytosis, and secrete inflammatory mediators. Hemocytes also infiltrate the CNS under stress conditions, contributing to neuroimmune surveillance and repair (3). The fat body, a multifunctional organ analogous to the mammalian liver and adipose tissue, acts as the primary site of systemic immunity and metabolic regulation. It synthesizes and secretes antimicrobial peptides (AMPs) and cytokine-like molecules in response to immune signals from both the CNS and hemocytes (8).

Neurodegenerative stress originating in the CNS triggers a cascade of inter-organ communication. This systemic signaling is mediated by humoral factors (e.g., AMPs, Upd cytokines), extracellular vesicles (including exosomes), and conserved pathways such as Toll, Imd, and JAK/STAT. These signals coordinate peripheral immune activation and feedback into the CNS, shaping the trajectory of neuroinflammation and degeneration (10, 11). Understanding this brain–peripheral immune axis in *Drosophila* provides a powerful framework for dissecting conserved neuroimmune mechanisms and identifying therapeutic targets relevant to human neurodegenerative diseases.

## 2 The neuroimmune landscape in *Drosophila melanogaster*

### 2.1 Overview of immune components: glia, hemocytes, and the fat body

In *D. melanogaster*, innate immunity relies on three specialized yet interdependent tissues: glial cells within the central nervous system, circulating hemocytes in the hemolymph, and the fat body as the systemic immune and metabolic hub. These components, as outlined in the Introduction (Section 1.3), coordinate localized defense and organism-wide inflammatory responses to neurodegenerative stimuli, providing a streamlined yet evolutionarily conserved framework for investigating neuroimmune interactions (3, 8, 10, 12).

Glial cells, which reside within the CNS and include cortex, ensheathing, and astrocyte-like subtypes, perform roles analogous to mammalian microglia and astrocytes, including debris clearance, synaptic remodeling, and neuroprotection. They respond to injury by activating conserved immune pathways such as Toll and JAK/STAT, making them central to brain-peripheral immune communication (10, 12).

Hemocytes are the primary circulating immune cells, functionally comparable to vertebrate macrophages and myeloid cells. They patrol the hemolymph, engage in phagocytosis, and initiate systemic responses. Under neurodegenerative conditions, hemocytes can infiltrate the CNS to modulate glial activity, contributing to inflammation and tissue repair (3, 8).

The fat body serves as the central metabolic and immune organ, analogous to the mammalian liver and adipose tissue. It is the principal site for the synthesis and secretion of systemic immune mediators, such as antimicrobial peptides (AMPs), Unpaired (Upd) cytokines and Eiger (a TNF-like ligand), which enable cross-talk between peripheral immunity and CNS-resident glia (8, 11).

This tripartite immune organization allows *Drosophila* to mount coordinated responses to neurodegenerative stress, making it a powerful model for dissecting conserved neuroimmune mechanisms and identifying therapeutic targets relevant to human disease.

### 2.2 Core innate immune signaling pathways in *Drosophila melanogaster*

**Figure 1:**
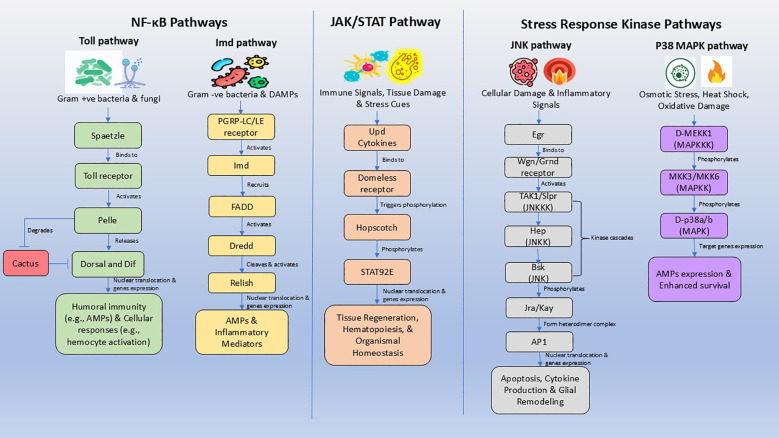
Core innate immune signaling pathways regulating neuroimmune crosstalk in *Drosophila melanogaster*. This schematic illustrates the major conserved innate immune signaling pathways involved in neuroimmune regulation in *Drosophila melanogaster*. The NF-κB pathways include the Toll pathway, activated primarily by Gram-positive bacteria and fungi via Spaetzle–Toll–Pelle signaling leading to Dorsal/Dif nuclear translocation, and the Imd pathway, activated by Gram-negative bacteria and damage-associated molecular patterns (DAMPs) through PGRP-LC/LE, Imd, FADD, and Dredd, culminating in Relish activation and antimicrobial peptide (AMP) expression. The JAK/STAT pathway, triggered by immune stress and tissue damage, is activated by Unpaired (Upd) cytokines binding to the Domeless receptor, leading to Hopscotch-mediated phosphorylation of STAT92E and transcriptional programs regulating tissue regeneration, hematopoiesis, and systemic immune homeostasis. In parallel, stress-response kinase pathways, including JNK and p38 MAPK, respond to cellular damage, oxidative stress, osmotic stress, and inflammatory cues. JNK signaling via Eiger–Wengen/Grindelwald receptors and the TAK1–Hep–Basket cascade regulates apoptosis, cytokine production, and glial remodeling, while the p38 MAPK pathway (D-MEKK1–MKK3/6–p38a/b) promotes stress adaptation and immune gene expression. Collectively, these pathways integrate central nervous system and peripheral immune signals, forming the molecular backbone of brain–peripheral immune crosstalk that influences neuroinflammation, glial activation, and neurodegeneration.

The innate immune system of *Drosophila* relies on a suite of evolutionarily conserved signaling pathways that detect microbial threats and endogenous stress, triggering transcriptional programs essential for host defense, tissue repair, and neuroimmune regulation. These pathways such as NF-κB (Toll and Imd) (13), JAK/STAT (8), and stress-activated kinases (JNK and p38 MAPK) (14) serve as molecular bridges between immune activation and neurodegenerative outcomes (**Figure 1**).

#### 2.2.1 NF-κB pathways: toll and Imd

The Toll and Imd pathways are the two canonical NF-κB signaling modules in *Drosophila*, each tuned to distinct microbial and stress cues. The Toll pathway is activated primarily by Gram-positive bacteria and fungi. Recognition occurs via the ligand Spaetzle, which binds the Toll receptor, initiating a cascade involving Pelle (a kinase) and Cactus (an IκB-like inhibitor). This leads to the nuclear translocation of NF-κB transcription factors Dorsal and Dif, which regulate genes involved in both humoral immunity (e.g., antimicrobial peptides) and cellular responses (**Figure 1**) (e.g., hemocyte activation) (8, 13).

The Imd pathway responds predominantly to Gram-negative bacteria and increasingly to damage-associated molecular patterns. It is initiated by PGRP-LC/LE receptors and signals through Imd, FADD, and Dredd (a caspase), culminating in the activation of Relish, a unique NF-κB factor with both DNA-binding and inhibitory domains. Relish drives the transcription of most AMPs and inflammatory mediators. Chronic Imd hyperactivation has been linked to neurodegenerative phenotypes and glial dysfunction (9, 10, 15).

#### 2.2.2 JAK/STAT pathway

The Janus Kinase/Signal Transducer and Activator of Transcription (JAK/STAT) pathway mediates systemic immune signaling and tissue regeneration. It is activated by Unpaired (Upd) cytokines like Upd1, Upd2, and Upd3 which are functionally analogous to mammalian IL-6 (**Figure 1**) (16). These ligands bind the transmembrane receptor Domeless (Dome), triggering phosphorylation by the Janus kinase homolog Hopscotch (Hop). The downstream effector STAT92E translocates to the nucleus and regulates genes involved in hematopoiesis, epithelial repair, and neuroimmune communication, including macrophage infiltration into the CNS and glial activation (16, 17). Beyond immunity, JAK/STAT signaling integrates nutritional status and stress cues, making it a key regulator of organismal homeostasis.

#### 2.2.3 Stress-response kinases: JNK and p38 MAPK

The JNK and p38 MAPK pathways are conserved stress-responsive cascades that link environmental and intracellular stress to immune activation. The p38 MAPK pathway, activated by kinases such as D-MEKK1 (homologous to mammalian MEKK4/MTK1), responds to osmotic stress, heat shock, and oxidative damage. It modulates AMP expression and enhances survival under hostile conditions (8, 14). The JNK pathway is triggered by cellular damage and inflammatory signals, regulating apoptosis, cytokine production, and glial remodeling. In neurodegenerative contexts, both JNK and p38 MAPK serve as central hubs that integrate stress signals with immune gene expression, contributing to chronic neuroinflammation and tissue degeneration (**Figure 1**) (8, 10, 14).

### 2.3 Comparison with mammalian orthologs

The innate immune system of *D. melanogaster* offers a powerful reductionist framework for dissecting conserved inflammatory signaling. Despite its simplicity, the fly retains core molecular modules such as NF-κB, JAK/STAT, and MAPK pathways that are evolutionarily conserved across metazoans. This conservation enables researchers to manipulate individual components (e.g., Relish, the fly homolog of NF-κB) in a tissue-specific manner to unravel their mechanistic roles in neurodegeneration, immune activation, and stress responses (**Figure 1**, **Table 1**) (9, 15, 18).

**Table 1:** Comparison of core innate immune pathway orthologs in *Drosophila* vs. mammals.

*Drosophila* component/pathway	Mammalian ortholog	Function in immunity/neuroinflammation	References
Toll	Toll-like Receptors (TLRs)	Pattern recognition; activation of NF-κB via Spaetzle–Toll–Pelle–Cactus cascade.	(9, 13, 18)
Imd Pathway	TNF Receptor (TNFR), IL-1 Receptor	Response to Gram-negative bacteria and DAMPs; activates Relish via Imd–FADD–Dredd signaling.	(8, 10, 15)
Relish (NF-κB factor)	p65/RelA (NF-κB), cRel	Transcription of immune genes (e.g., AMPs); implicated in chronic neuroinflammation.	(7, 15)
Upd/Upd2/Upd3	Interleukin-6 (IL-6) family cytokines	Systemic inflammatory signaling; activates JAK/STAT via Dome receptor.	(3, 8, 17)
Domeless (Dome)	GP130/IL-6 Receptor	Transmembrane receptor for Upd cytokines; initiates Hop–STAT92E signaling.	(3, 12)
Draper	MEGF10, Jedi-1, CED-1 (C. elegans)	Phagocytic receptor for apoptotic debris and synaptic pruning; glial clearance in neurodegeneration.	(19, 20)

In contrast, mammalian systems present substantial complexity. Multiple paralogs of NF-κB (e.g., p65/RelA, c-Rel, RelB, p50, p52), diverse cytokine receptors, and overlapping signaling cascades introduce functional redundancy, making it difficult to isolate the contribution of a single pathway. Moreover, the presence of adaptive immunity and specialized glial subtypes further complicates interpretation (5, 6, 18). By leveraging the genetic tractability and modular organization of *Drosophila*, researchers can perform high-resolution analyses of immune–neurodegenerative interactions. The following table outlines key molecular orthologs between *Drosophila* and mammals, emphasizing the conserved signaling toolkit that underpins innate immunity across species.

## 3 Brain-resident immune cells and glial activation

### 3.1 Functional parallels with mammalian microglia and astrocytes

In *D. melanogaster*, glial cells play a central role in maintaining neural integrity, performing functions that closely mirror those of mammalian microglia and astrocytes. These brain-resident immune cells are responsible for monitoring neural activity, modulating synaptic architecture, and clearing cellular debris, thereby ensuring circuit stability and neuroprotection (17, 18).

Among the glial subtypes, ensheathing glia and cortex glia are particularly relevant to neuroimmune interactions.

- Ensheathing glia envelops the synaptic neuropil, such as the olfactory antennal lobe, and act as the primary phagocytes of the CNS. Their role in clearing apoptotic neurons and damaged synapses is functionally analogous to mammalian microglia, especially during neurodegenerative stress and developmental pruning (19, 20).
- Cortex glia, which surround neuronal cell bodies, are essential for engulfing dying neurons during development and for maintaining homeostatic balance in the adult brain. They also contribute to experience-dependent plasticity and glial remodeling in response to environmental stimuli (17, 21).

These glial populations are regulated by conserved signaling pathways such as Draper-mediated phagocytosis (19), JAK/STAT (17), and NF-κB (15), which coordinate immune responses and structural refinement. Their ability to respond to both intrinsic and extrinsic cues make them indispensable for studying neuroimmune crosstalk and glial dysfunction in neurodegeneration.

### 3.2 Mechanisms of phagocytosis and debris clearance

In *D. melanogaster*, the glial phagocytic machinery is orchestrated primarily by the evolutionarily conserved receptor Draper, a homolog of mammalian MEGF10 and Jedi-1, and the *Caenorhabditis elegans* receptor CED-1. Draper is indispensable for the recognition and engulfment of neuronal debris, apoptotic cells, and degenerating synapses, positioning it as a central regulator of CNS immune surveillance and remodeling (17, 19). Recent findings have expanded Draper’s role beyond acute injury responses. Ensheathing glia, which surround synaptic neuropil regions such as the antennal lobe, express Draper constitutively. While its expression is upregulated following axonal injury, Draper also mediates ongoing synaptic refinement in the uninjured brain. This includes regulation of presynaptic bouton size, terminal arbor morphology, and synaptic content, underscoring its role in experience-dependent plasticity and circuit homeostasis (19, 22).

Under conditions of chronic neurodegenerative stress, damaged neurons release damage-associated molecular patterns that activate Draper-mediated phagocytosis. Although this mechanism is essential for maintaining neural integrity, it can become pathologically overactivated in the presence of neurotoxic aggregates such as Tau or Amyloid-β (Aβ), leading to excessive synaptic pruning and early synaptotoxicity. This mirrors microglial-driven synapse loss observed in mammalian models of AD and Tauopathies (18, 22). Together, these insights position Draper as a mechanistic bridge between homeostatic glial function and pathological neurodegeneration, making it a promising target for therapeutic modulation in fly-based models of CNS disease.

### 3.3 Activation of local innate pathways (NF-κB/Relish) and ROS

In *Drosophila melanogaster*, glial activation under neurodegenerative conditions involves the initiation of conserved innate immune pathways, most notably the NF-κB signaling cascade. The transcription factor Relish, a *Drosophila*-specific NF-κB homolog, is upregulated in glial cells in response to stress and damage, mirroring the inflammatory responses observed in mammalian microglia and astrocytes (8, 18). While Relish primarily drives the expression of immune-responsive genes such as AMPs, emerging evidence suggests its involvement in non-canonical cell death pathways. Under chronic neurodegenerative stress, sustained Relish activation may shift from a protective immune role to a pathogenic executor, promoting glial dysfunction and neuronal loss. This transition reflects a critical threshold where immune signaling becomes maladaptive, contributing to synaptic degeneration and neuronal vulnerability (10, 18).

This inflammatory cascade is tightly coupled with oxidative stress, particularly the accumulation of ROS. Neurodegeneration-associated genes such as LRRK2 and DJ-1 have been shown to impair mitochondrial function, leading to elevated ROS production. ROS not only damage cellular components but also disrupt mitochondrial dynamics, favoring fission over fusion, which exacerbates mitochondrial fragmentation and dysfunction (10, 11). The resulting ROS-inflammation feedback loop amplifies glial immune activation, creating a self-perpetuating cycle of neurotoxicity. This mechanistic link between mitochondrial failure, oxidative stress, and innate immune overactivation provides a compelling framework for understanding early synaptotoxic events in neurodegenerative diseases such as PD and AD (11).

## 4 Peripheral immune components and systemic crosstalk

### 4.1 Hemocyte functions and CNS communication

In *D. melanogaster*, hemocytes serve as the primary circulating immune cells, analogous to mammalian macrophages and myeloid lineages. These cells are essential not only for phagocytosing pathogens and apoptotic debris, but also for initiating and coordinating humoral immune responses through the secretion of antimicrobial peptides and cytokine-like molecules (8). Recent studies have revealed a direct and dynamic communication axis between the CNS and peripheral hemocytes. This neuroimmune dialogue is mediated by acetylcholine, a neurotransmitter released by neurons and glia, which acts on nicotinic acetylcholine receptors (nAchRs) expressed on hemocytes. Activation of these receptors modulates the Toll pathway in hemocytes, influencing their inflammatory profile and population dynamics (13).

This cholinergic signaling mechanism establishes a rapid and evolutionarily conserved interface between neural activity and immune regulation. It demonstrates that the peripheral immune state is not autonomous, but rather under continuous modulation by CNS-derived signals. Such findings underscore the importance of neuroimmune integration in maintaining systemic homeostasis and responding to neurodegenerative stress (13). Importantly, this CNS-to-hemocyte communication is bidirectional. Hemocytes can infiltrate the brain under stress conditions, particularly during neurodegeneration, where they contribute to debris clearance, cytokine release, and glial modulation. This infiltration is often mediated by JAK/STAT signaling, which is activated by CNS-derived Upd cytokines and facilitates macrophage-like behavior in hemocytes (3).

Such findings underscore a dynamic neuroimmune axis, where peripheral immune tone is shaped by neural activity, and immune cells reciprocally influence CNS integrity. This axis is evolutionarily conserved and mirrors mammalian neuroimmune interactions, where vagal signaling, cytokine feedback, and blood–brain barrier modulation coordinate systemic and central inflammation.

### 4.2 Fat body–brain signaling: cytokine analogs

In *D. melanogaster*, the fat body serves as a multifunctional organ integrating metabolic regulation, immune surveillance, and endocrine signaling. Analogous to the mammalian liver and adipose tissue, it secretes conserved cytokine-like molecules that mediate systemic responses to stress, infection, and neurodegeneration. These humoral signals enable bidirectional communication between peripheral tissues and the CNS, shaping organism-wide inflammatory tone and metabolic adaptation (8).

#### 4.2.1 The Upd/JAK/STAT axis

The Upd family such as Upd1, Upd2, and Upd3 comprises cytokine analogs structurally and functionally similar to mammalian IL-6. These ligands activate the JAK/STAT pathway by binding to the transmembrane receptor Dome, initiating phosphorylation via the Janus kinase homolog Hop and downstream transcriptional activation by STAT92E (8, 17).

Among these, Upd3 has emerged as a key mediator in neurodegenerative models. Expression of neurotoxic proteins such as Aβ42 in the CNS triggers the release of Upd3, which then circulates systemically to activate JAK/STAT signaling in skeletal muscle. This activation leads to mitochondrial dysfunction, impaired energy metabolism, and progressive motor deficits, even in the absence of direct neural damage (17).

This mechanism highlights how brain-derived inflammatory signals can propagate through peripheral tissues, offering a conserved explanation for non-motor symptoms such as cachexia and muscle weakness observed in human neurodegenerative diseases. Upd2, secreted by the fat body, also plays a role in developmental and regenerative processes, such as guiding tracheal stem cell migration, further underscoring the systemic reach of fat body-derived cytokines (16).

#### 4.2.2 Eiger: the TNF-α analog

Eiger, the *Drosophila* homolog of Tumor Necrosis Factor-α (TNF-α), is a fat body-derived cytokine that modulates growth, metabolism, and inflammatory feedback to the CNS via the Wengen receptor. Its expression is sensitive to nutritional status and gut-derived lipid signals, linking peripheral metabolic stress to central regulation of insulin secretion and neuroimmune tone (23, 24).

Beyond metabolic roles, Eiger contributes to neurodegenerative signaling by activating JNK-dependent pathways involved in cell death, glial activation, and synaptic remodeling, especially under chronic stress or protein aggregation (10, 23). Together with Upd cytokines, Eiger forms a fat body–brain axis that reinforces the concept of whole-organism inflammatory coordination in neurodegeneration.

### 4.3 Role of gut microbiota and the gut–brain–immune axis

The gut–brain–immune axis represents a dynamic communication network where signals from the intestinal microbiota and immune system significantly influence CNS function. *Drosophila melanogaster* has emerged as a powerful model for dissecting this axis due to its genetic tractability and simplified immune architecture (23–25). Recent research has shown that immune dysregulation originating in the gut can independently trigger neurodegenerative changes, even in the absence of classical protein aggregates like Aβ or tau. A key player in this process is Pirk, a negative regulator of the Imd/NF-κB pathway. Loss of Pirk leads to hyperactivation of NF-κB signaling in the gut epithelium, resulting in chronic inflammation and systemic immune imbalance (25). This gut-derived immune stress propagates to the brain, where it induces glial activation, oxidative stress, and neuronal dysfunction, ultimately impairing motor behavior and lifespan. The study demonstrates that intestinal immune homeostasis is essential for maintaining CNS integrity, and that microbiota-driven inflammation can act as a primary driver of neurodegeneration (24, 25).

Crucially, the microbiota’s influence is not limited to driving generalized inflammation; it also directly modulates the severity of established neurodegenerative proteinopathies. In transgenic *Drosophila* models that express the core pathological human proteins, the gut microbiota acts as a critical modifier. For instance, in PD (α-synuclein) models, flies overexpressing human α-synuclein (α-Syn) in the CNS show exacerbated locomotor deficits and neuronal loss due to gut microbial dysbiosis (26). Similarly, in AD (Aβ and Tau) models, alterations in the gut microbiota profile worsen neurological function. This mechanism involves gut-derived bacterial metabolites, such as acetate and lactate, which are produced by common fly commensals and have been shown to influence CNS functions by modulating glial cell activation and Tau protein dynamics in *Drosophila* ensheathing glia (27). These data confirm that the gut microbiota is a direct and potent factor in dictating the neurotoxicity induced by the signature misfolded proteins of NDs.

### 4.4 Temporal hierarchy of immune dysregulation

Studies in *D. melanogaster* have revealed a temporal hierarchy in immune-driven neurodegeneration, with the gut emerging as the earliest site of pathological initiation. Central to this process is Pirk, a negative regulator of the Imd/NF-κB pathway. Loss of *pirk* leads to chronic immune hyperactivation, resulting in age-dependent neurological phenotypes including reduced locomotion, altered sleep patterns, and an increased burden of brain lesions (25). Using tissue-specific RNA interference (RNAi), researchers demonstrated that gut-specific knockdown of pirk particularly in intestinal stem cells induces early-onset neurodegenerative symptoms, implicating the intestinal immune system as a primary driver. In contrast, glia-specific pirk knockdown results in delayed neurological decline, suggesting that CNS inflammation is a secondary contributor to the overall pathology (24, 25).

This temporal stratification underscores the causal role of peripheral immune dysregulation, particularly from the gut ecosystem, in initiating and accelerating neurodegeneration. It aligns with broader findings that intestinal barrier dysfunction, microbiota imbalance, and NF-κB overactivation can propagate systemic inflammation and CNS damage (28, 29). These insights reinforce the concept that neurodegeneration is a multi-organ process, where early immune stress in the gut sets the stage for later CNS pathology, even in the absence of classical protein aggregates like Aβ or tau.

### 4.5 Microbiota and AMP mediators

The early onset of neurodegenerative symptoms in *D. melanogaster* pirk mutants is driven by two converging factors: intestinal dysbiosis and chronic overexpression of AMPs. Loss of *pirk*, a negative regulator of the Imd/NF-κB pathway, results in persistent immune activation in the gut, altering microbial composition and amplifying inflammatory signaling. Experimental evidence shows that rearing pirk mutants under axenic (germ-free) conditions significantly rescues neurological and sleep-related defects, confirming the causative role of the microbiota. Furthermore, genetic knockout of the AMP gene *AttacinD (AttD)* mitigates behavioral impairments, implicating AMP toxicity as a direct contributor to CNS decline (25).

AMPs such as AttD, while essential for pathogen defense, can become neurotoxic when chronically expressed, especially in the context of gut immune dysregulation. These peptides may act systemically, crossing tissue barriers or activating glial NF-κB signaling, leading to oxidative stress, synaptic dysfunction, and neuronal loss (17, 23). Additional studies have shown that microbiota composition influences AMP expression and neuroimmune tone. For example, commensal bacteria regulate intestinal stem cell renewal, barrier integrity, and immune tolerance, while dysbiosis promotes inflammatory cascades that propagate to the brain (28–30).

These findings position the therapeutic window for restoring immune balance early in disease progression, specifically within the peripheral/gut ecosystem. Targeting microbiota composition and AMP regulation may offer a strategy to delay or prevent CNS pathology, reinforcing the gut’s role as a primary site of neuroimmune initiation.

## 5 Inflammation in *Drosophila* models of neurodegeneration

### 5.1 α-Synuclein models of Parkinson’s disease

*D. melanogaster* expressing human α-Syn in dopaminergic (DA) neurons provides a robust and genetically tractable model for PD. These flies recapitulate hallmark features of PD, including progressive DA neuron loss, locomotor deficits, and age-dependent neurodegeneration (10).

Longitudinal transcriptomic and proteomic profiling of α-Syn-expressing flies has revealed accelerated activation of innate immune pathways, notably those resembling neutrophil degranulation, platelet activation, and NF-κB signaling processes also observed in human PD patients (31). This upregulation occurs earlier and more intensely than in age-matched controls, supporting the hypothesis that α-Syn toxicity accelerates inflammaging, the chronic low-grade inflammation associated with aging (10, 31).

Importantly, this chronic inflammatory state imposes a molecular burden on neurons. Multi-omics analyses revealed early and significant downregulation of pathways essential for neuronal maintenance, including those governing synaptic function, protein translation, and mitochondrial energy metabolism. These findings suggest that inflammation not only damages neurons externally but also divert cellular resources away from repair and homeostasis, thereby compromising neuronal resilience and accelerating degeneration (31, 32).

This model underscores the causal role of neuroinflammation in PD progression and highlights the utility of *Drosophila* for dissecting the temporal and molecular dynamics of α-Syn-induced pathology.

### 5.2 APP/BACE1 and tau transgenic models of Alzheimer’s disease

*D. melanogaster* has proven to be a powerful model for dissecting the molecular and immunological underpinnings of AD. Transgenic flies expressing human Aβ42 or Tau recapitulate key pathological features, including progressive neurodegeneration, behavioral impairments, and immune activation. Accumulating Aβ peptides have been shown to act as potent activators of innate immune responses in both the CNS and peripheral tissues, triggering chronic inflammation and glial reactivity (10, 33).

Overexpression of the *Drosophila* APP homolog, Appl, in combination with human Tau, leads to severe adult phenotypes, including disrupted axonal transport, synaptic dysfunction, and reduced lifespan. These phenotypes mirror the cellular transport deficits and tauopathy-associated degeneration observed in mammalian AD models (27, 34).

Mechanistic studies have further demonstrated that modulating intestinal innate immune signaling particularly through the Imd/NF-κB pathway can ameliorate Aβ-induced behavioral and survival impairments, highlighting the systemic link between gut immune tone and CNS pathology (25, 27). This supports the emerging view that peripheral immune dysregulation, especially from the gut ecosystem, plays a causal role in accelerating neurodegeneration, even in the absence of direct neural insults.

Moreover, interactions between Tau and α-Syn in dual transgenic models have been shown to augment neurotoxicity, suggesting that protein aggregation synergy exacerbates inflammatory signaling and neuronal vulnerability (34).

### 5.3 Evidence linking chronic inflammation to neuronal integrity

A pivotal demonstration of causality between chronic inflammation and neurodegeneration comes from *D. melanogaster* models where innate immunity is dysregulated independently of proteinopathy. In particular, the pirk knockout model which lacks a negative regulator of the Imd/NF-κB pathway exhibits persistent immune activation, elevated AMP expression (e.g., *AttacinD*), and gut microbiota alterations. These combined factors directly lead to neurological phenotypes, including measurable brain lesions, motor deficits, and sleep disturbances (25).

This model confirms that chronic innate immune signaling alone is sufficient to drive age-dependent neurodegeneration, even in the absence of classical protein aggregates like Aβ or Tau. It validates dysregulated immunity as an independent and actionable therapeutic target, shifting the focus from neuron-centric interventions to systemic immune modulation. These findings align with broader evidence that gut-derived inflammation, AMP toxicity, and NF-κB hyperactivation can compromise neuronal integrity and accelerate CNS decline (17, 27).

### 5.4 Oxidative and mitochondrial stress interplay

Neuroinflammation, mitochondrial dysfunction, and oxidative stress form a tightly interlinked pathological triangle that drives neurodegeneration. In *D. melanogaster* models of PD, mutations in conserved genes such as LRRK2 and DJ-1 disrupt mitochondrial dynamics, impair proteostasis, and elevate ROS production (10).

The accumulation of ROS triggers oxidative stress, which promotes mitochondrial fission and inhibits fusion-related proteins such as Mitofusin and Opa1, further destabilizing mitochondrial architecture (35). This chronic state of mitochondrial fragmentation and energy failure initiates and sustains neuroinflammatory signaling, recruiting glial cells and peripheral immune effectors into a self-reinforcing feedback loop (10, 35).

These findings underscore the mechanistic convergence of oxidative damage, mitochondrial collapse, and immune activation, validating this triad as a central axis in PD pathology and a promising target for therapeutic intervention (**Table 2**).

**Table 2:** Summary of *Drosophila* neurodegenerative models: immune signatures, inter-organ crosstalk, and outcomes.

Model type	Genetic manipulation/trigger	Key immune signatures	Inter-organ crosstalk observed	Observed outcomes	References
Parkinson’s Disease (PD)	α-Syn overexpression, Pink1/parkin mutants	Upd3/IL-6 analogs, NF-κB (Relish), Imd pathway activation	Gut–brain axis via Upd3; systemic inflammation affecting CNS and peripheral motor circuits	Dopaminergic neuron loss, locomotor decline, systemic inflammation	(36, 58, 59)
Alzheimer’s Disease (AD)	Aβ42 expression, Tau mutants	Toll pathway activation, Draper-mediated glial response	Glial–neuronal remodeling; immune signals modulate synaptic architecture and memory circuits	Synaptic degeneration, memory impairment, glial engulfment of neuropil	(12, 19, 26, 34, 37)
ALS-like Model: TDP-43/FUS	TDP-43, FUS transgenics	Elevated AMPs, MEK/ERK pathway, Imd/Toll dysregulation	Peripheral immune activation influences motor neuron integrity and lifespan	Motor dysfunction, reduced lifespan, neuroinflammation	(11, 60, 61)
ALS-like Model: SOD1	Pan-neuronal mutant SOD1 expression	Early NF-κB activation, chromosomal instability	Systemic inflammation and genomic stress affecting motor circuits	Motor decline, neuroinflammation, reduced survival	(11, 61)
Inflammaging/Aging Model	Natural aging, Withaferin A intervention	AMP specificity loss, Upd3 elevation, oxidative stress markers	Age-related immune tone affects gut–brain signaling and systemic stress response	Reduced lifespan, increased immune tone, phase-specific protection with phytochemicals	(42, 59, 62)
Diet-Induced Neurodegeneration	High-sugar/high-fat diet	Insulin resistance, systemic inflammation, TOR signaling	Metabolic–neural axis; diet-induced inflammation alters glial function and CNS integrity	Accelerated neurodegeneration, glial dysfunction, metabolic stress	(26, 57)
Environmental Toxin Model	Paraquat exposure	NF-κB activation, Pirk loss, ROS accumulation	Peripheral toxin exposure triggers CNS immune activation and dopaminergic vulnerability	Dopaminergic loss, innate immune hyperactivation, motor decline	(58, 59)
Microbiome–Neuroimmune Axis Model	Predator stress, microbiome manipulation	TOR–Upd3 axis, AMP modulation, gut–brain cytokine signaling	Gut microbiome modulates TOR signaling and aversive memory via immune–neural crosstalk	Altered aversive memory, immune-mediated behavioral plasticity	(26, 56, 63)

## 6 Molecular pathways mediating brain–peripheral crosstalk

### 6.1 Conserved stress kinases as inter-tissue hubs

The JNK and p38 MAPK pathways serve as evolutionarily conserved hubs that integrate cellular damage, environmental stress, and cytokine signaling across tissues. In *D. melanogaster*, JNK activation in response to injury or stress induces the expression of Upd cytokines, particularly Upd3, which initiates local regenerative programs and triggers a systemic innate immune response (17). This response is further amplified by secondary Upd production in the fat body, establishing a feedback loop that links damaged tissues to peripheral immune activation (10, 17).

Parallel to JNK, the p38 MAPK pathway, regulated by upstream kinases such as D-MEKK1, orchestrates responses to environmental and neuroendocrine stressors. Recent studies show that paternal restraint stress can activate a p38–MEKK1–dATF-2 axis, leading to Upd3 secretion and systemic cytokine signaling (14). These cytokines engage STAT-dependent transcriptional programs in distant tissues such as muscle, completing a brain–periphery communication loop that modulates inflammation and tissue homeostasis (35, 36).

Together, these pathways illustrate how stress-responsive kinases act as molecular bridges between neuronal stress and peripheral immune tone, reinforcing the concept of inter-organ signaling in neurodegenerative disease progression.

### 6.2 Humoral signaling and extracellular vesicles

In *D. melanogaster*, humoral factors such as cytokines (Upd, Eiger) and hormonal peptides like Dilp8 act as key mediators of long-range neuroimmune communication. These soluble signals coordinate responses between the brain, gut, and peripheral immune tissues. For instance, damage-induced Upd cytokines activate the JAK/STAT pathway, triggering a systemic immune response that promotes the proliferation of circulating macrophages and modulates tissue regeneration (10, 17).

Recent work has also highlighted the role of Dilp8, a stress-responsive insulin-like peptide, in gut–brain signaling, where it regulates insulin secretion and developmental timing via neuroendocrine feedback loops (23). These findings reinforce the concept that soluble peptides and cytokines serve as primary messengers in inter-organ communication during stress and neurodegeneration.

Beyond soluble factors, Extracellular Vesicles (EVs), especially exosomes have emerged as conserved vehicles for intercellular signaling. Exosomes carry proteins, lipids, and nucleic acids, and can modulate immune responses by either stimulating or suppressing inflammation, depending on their cellular origin and molecular cargo (8, 37). While direct evidence of exosome-mediated signaling between glia, neurons, and peripheral immune cells in *Drosophila* neurodegeneration is still developing, this mechanism is strongly supported by mammalian studies and represents a plausible route for transferring neurotoxic or inflammatory signals across the blood–brain barrier analog (37).

### 6.3 Transcriptomic and proteomic evidence from fly models

High-resolution transcriptomic and proteomic profiling in *Drosophila melanogaster* has illuminated the temporal and cell-type-specific dynamics of neuroimmune interactions, revealing that the fate of innate immunity is not uniform across neurodegenerative diseases but instead reflects the primary cellular pathology.

In AD models expressing human Aβ or Tau, glial cells exhibit robust transcriptional activation of the Draper-mediated phagocytic machinery, a homolog of mammalian MEGF10 (38). However, despite this activation, functional clearance of protein aggregates is inefficient, leading to chronic glial stress, oxidative damage, and non-cell-autonomous neurotoxicity (11, 27). This suggests that glial immune activation, while transcriptionally robust, becomes functionally decoupled from effective proteostasis, contributing to disease progression.

In contrast, longitudinal studies in α-Syn-expressing PD models reveal a distinct immune trajectory. Time-course transcriptomic analyses show early and sustained upregulation of innate immune pathways, including antimicrobial peptides and NF-κB targets, alongside a concurrent repression of neuronal maintenance programs such as protein translation, synaptic function, and mitochondrial energy metabolism (31). This dual molecular shift suggests that chronic immune activation imposes a metabolic burden, forcing neurons to divert resources away from repair and survival, thereby compromising their resilience.

Moreover, cell-type-specific transcriptomic comparisons, such as those conducted in NPFR neurons, reveal that the impact of inflammatory stressors is modulated by the proteomic identity and functional specialization of the target neuron (39). Neurons with distinct proteomic landscapes exhibit differential vulnerability to immune insults, highlighting the importance of neuronal heterogeneity in shaping disease outcomes.

Together, these insights underscore that while innate immune activation is a shared feature across neurodegenerative models, its functional consequences diverge ranging from glial clearance failure in AD to neuronal metabolic collapse in PD depending on the molecular context and cellular targets.

## 7 Therapeutic modulation of neuroimmune crosstalk

*D. melanogaster* offers a genetically tractable and cost-effective platform for screening neuroprotective compounds and validating therapeutic targets that disrupt the neuroimmune feedback loop. Its short lifespan and conserved molecular pathways make it ideal for evaluating life-stage-specific drug efficacy in ND models.

### 7.1 Natural compounds and life-stage dependent efficacy

#### 7.1.1 Curcumin: the closing therapeutic window

Curcumin, a plant-derived polyphenol with well-established anti-inflammatory and antioxidant properties, has shown promising results in *Drosophila* models of PD. In young adult flies, curcumin treatment led to restoration of brain dopamine levels, improved locomotor function, and reduced neurodegeneration, confirming its neuroprotective potential (40).

However, this efficacy was found to be strictly dependent on the life stage. In older adult flies representing the vulnerable transition phase when late-onset PD typically manifests curcumin failed to confer protection, despite its continued ability to mitigate oxidative stress (40, 41). This suggests that aging-related molecular decline, rather than drug potency, limits therapeutic success.

Mechanistic studies revealed that the aged brain exhibits depleted expression of key neuroprotective regulators, including Mfn2 (a mitochondrial fusion protein), dFOXO and GADD45 (stress response mediators), and Phase II antioxidant enzymes such as CncC and Prx. These molecules are essential for maintaining neuronal integrity, and their loss compromises the cell’s ability to respond to therapeutic interventions (41).

This insight carries significant clinical implications for late-onset neurodegenerative diseases, the therapeutic window for agents that rely on intrinsic resilience mechanisms may close as aging and chronic inflammation erode the molecular infrastructure required for repair. Future drug development must therefore consider biological age and disease stage, potentially shifting toward therapies that restore resilience pathways rather than merely neutralizing toxic aggregates.

#### 7.1.2 Withaferin A: systemic and barrier protection

Withaferin A (WA), a steroidal lactone derived from *Withania somnifera*, has demonstrated multifaceted geroprotective effects in *D. melanogaster*. WA treatment significantly extends median and maximum lifespan, improves locomotor function, and enhances intestinal barrier integrity, particularly in aged flies, a critical factor in mitigating systemic inflammation and neurodegeneration (42).

Mechanistically, WA upregulates key genes involved in antioxidant defense and DNA damage response, including PrxV, Gadd45, and Ku80, thereby reinforcing cellular resilience against oxidative and genotoxic stress. These molecular effects are complemented by WA’s ability to modulate NF-κB signaling, reduce ROS accumulation, and suppress age-associated immune activation, positioning it as a promising candidate for neuroimmune modulation (42).

Importantly, the restoration of gut barrier function by WA supports the emerging view that the gut–immune axis plays a pivotal role in triggering neurodegenerative cascades. By simultaneously reducing systemic oxidative stress and reinforcing peripheral immune barriers, WA exemplifies the therapeutic potential of dual-action phytochemicals in aging and neurodegeneration (10, 42).

#### 7.1.3 Resveratrol: sirtuin activation and mitochondrial resilience

Resveratrol (RSV), a natural stilbene polyphenol found in grapes and berries, has emerged as a potent modulator of longevity and neuroprotection in *D. melanogaster*. Its therapeutic efficacy is largely attributed to the activation of sirtuin signaling, particularly dSir2, the fly homolog of mammalian SIRT1, which plays a central role in mitochondrial biogenesis, stress resistance, and metabolic regulation (43).

In *Drosophila* models of neurodegeneration, RSV treatment has been shown to extend lifespan, improve climbing ability, and reduce dopaminergic neuronal loss, especially under oxidative stress conditions induced by paraquat or rotenone exposure. These benefits are mediated through enhanced AMPK signaling, increased mitochondrial membrane potential, and upregulation of antioxidant enzymes such as SOD2 and Catalase, which collectively preserve neuronal integrity (44).

Importantly, RSV also modulates inflammatory tone by suppressing JNK and Relish/NF-κB pathways, thereby reducing age-associated immune activation and glial overactivation (43). This dual action boosting mitochondrial resilience while dampening neuroinflammation positions RSV as a promising candidate for early-stage intervention in neurodegenerative disorders.

However, similar to curcumin, RSV’s efficacy appears to be life-stage dependent. Studies indicate diminished neuroprotective outcomes in aged flies, likely due to reduced sirtuin expression and compromised mitochondrial dynamics, suggesting that timing of intervention is critical for maximizing therapeutic benefit (44, 45).

#### 7.1.4 *Cordyceps* extracts: mitochondrial revitalization and immune modulation

*Cordyceps* spp., a genus of entomopathogenic fungi traditionally used in East Asian medicine, has gained attention for its neurotherapeutic potential in aging and neurodegenerative disorders. Extracts from *C. sinensis* and *C. militaris* contain bioactive compounds such as cordycepin, polysaccharides, and adenosine analogs, which exert antioxidant, anti-inflammatory, and mitochondria-enhancing effects (46).

In *D. melanogaster*, *Cordyceps* supplementation has been shown to extend lifespan, improve climbing ability, and reduce age-associated oxidative stress, particularly in models exposed to neurotoxins like paraquat or rotenone. These effects are mediated through upregulation of AMPK, PGC-1α, and SOD2, promoting mitochondrial biogenesis, energy homeostasis, and ROS clearance (47).

*Cordyceps* extracts also modulate the innate immune response in flies by downregulating Relish/NF-κB signaling, thereby reducing chronic inflammation and glial activation, hallmarks of neurodegeneration. Additionally, studies suggest improvements in gut barrier integrity and microbial homeostasis, reinforcing the gut–brain–immune axis as a therapeutic target (47, 48).

Importantly, *Cordyceps* appears to retain efficacy across multiple life stages, offering both preventive and restorative benefits. This distinguishes it from compounds like curcumin and resveratrol, whose effects are often limited to early or mid-life intervention windows.

### 7.2 Dopamine agonist nanocomposites

The *D. melanogaster* PD model has proven instrumental in advancing drug delivery systems aimed at overcoming the limitations of conventional dopamine agonists. Agents such as bromocriptine and levodopa, while clinically effective, suffer from poor pharmacokinetics, short half-life, and adverse side effects including dyskinesia, sleep disturbances, and peripheral toxicity (49). These drawbacks necessitate the development of targeted delivery platforms that enhance therapeutic efficacy while minimizing systemic burden.

Recent research has leveraged the *Drosophila* PD model to screen dopamine agonist nanocomposites, such as bromocriptine–alginate nanocomposites (BANC), which encapsulate the drug within a biocompatible polymer matrix. This formulation improves drug stability, controlled release, and CNS penetration, leading to enhanced dopaminergic neuron survival, restored locomotor function, and reduced oxidative stress in transgenic flies expressing human α-Syn (50).

Parallel studies in mammalian models have validated this approach. For instance, a Lf-DA-MSN-PF127 nanocomposite comprising lactoferrin, dopamine, mesoporous silica nanoparticles, and Pluronic F127 demonstrated efficient dopamine delivery, mitochondrial protection, and behavioral recovery in rat models of PD, confirming the translational potential of nanocarrier systems (51).

These findings underscore the utility of *Drosophila* as a preclinical screening platform for evaluating formulation efficacy, neuroprotective outcomes, and side effect mitigation prior to mammalian validation. The integration of nanotechnology with fly genetics offers a scalable and mechanistically rich pipeline for symptomatic and disease-modifying therapies in neurodegeneration.

### 7.3 Genetic modulation of immune pathways

Genetic manipulation in *D. melanogaster* has provided compelling evidence for the therapeutic validity of immune targets in managing systemic inflammation and neurodegeneration. Disruption of the JAK/STAT signaling pathway, particularly through mutations in core components such as the cytokine ligand Upd3 or its receptor Dome, significantly reduces systemic inflammatory responses and prevents peripheral defects including muscle mitochondrial dysfunction and metabolic collapse (52).

In parallel, studies on NF-κB signaling have revealed that chronic activation of the Imd pathway, a key innate immune cascade in flies, leads to age-dependent neurological decline, sleep disruption, and brain lesions. Loss-of-function mutations in Pirk, a negative regulator of the Imd/NF-κB pathway, exacerbate these phenotypes. However, genetic suppression of Imd signaling or rearing flies under axenic (germ-free) conditions rescues locomotor and cognitive deficits, confirming that peripheral immune tone directly influences neurodegenerative outcomes (25).

Interestingly, cross-species insights from crustacean models reveal that MAP3K15 (ASK3), a mitogen-activated protein kinase, facilitates viral gene expression by activating a Dorsal–CC-CL–STAT axis, alongside the JNK/P38 pathway. This cascade promotes immune evasion and viral replication, but its inhibition via SDK1 or RNAi significantly improves host survival (53).

These genetic studies reinforce the concept that targeting peripheral immune regulators whether through cytokine signaling, NF-κB suppression, or MAPK inhibition can restore systemic and neurological homeostasis. They also emphasize the need to consider tissue-specific immune dynamics and microbiota interactions when designing interventions for chronic inflammatory and neurodegenerative conditions.

### 7.4 Prospective drug screening pipelines

The *D. melanogaster* system offers a powerful platform for preclinical drug screening, particularly for evaluating optimized drug delivery systems that address the pharmacokinetic limitations of conventional therapeutics. Its genetic tractability, rapid life cycle, and conserved neurochemical pathways make it ideal for testing formulations in neurodegenerative contexts (51).

For example, dopamine agonists commonly used in PD such as bromocriptine and levodopa often suffer from poor bioavailability, short half-life, and systemic side effects (49). These limitations have prompted the development of nanocomposite-based delivery systems that enhance drug stability, target specificity, and CNS penetration.

Recent studies using *Drosophila* PD models have demonstrated the efficacy of BANC in improving locomotor function, dopaminergic neuron survival, and oxidative stress resistance, compared to free drug administration (50). These findings validate the fly model as a screening pipeline for nanotherapeutics and highlight its translational relevance.

Moreover, precision medicine approaches using *Drosophila* have enabled the dissection of gene–environment interactions, allowing researchers to test how genetic susceptibility modulates drug response (53). This is particularly valuable for stratifying therapeutic efficacy across life stages, genotypes, and environmental exposures.

## 8 Translational insights: from *Drosophila* to mammalian neuroimmunology

### 8.1 Conserved signaling molecules and functional orthologs

The most significant translational value of *Drosophila melanogaster* models lies in the deep evolutionary conservation of innate immune signaling pathways. Mechanistic insights into NF-κB (Relish), JAK/STAT (Upd–Dome–Stat92E), and phagocytic clearance (Eater, NimC1) have proven highly transferable to mammalian systems, where their orthologs regulate inflammation, tissue homeostasis, and neuroimmune crosstalk (10).

A particularly compelling example is the discovery that central nervous system–derived Upd3 cytokine in flies can induce peripheral mitochondrial dysfunction, especially in muscle tissue. This mirrors how IL-6 family cytokines in mammals released from the brain during neurodegeneration can trigger systemic immune activation and non-motor symptoms in disorders like PD and AD (36). The *Drosophila* model thus provides a mechanistic blueprint for understanding how central pathology drives peripheral decline, reinforcing the need to target systemic inflammatory tone alongside CNS-specific interventions.

These findings strongly validate the therapeutic strategy of modulating peripheral immune pathways such as NF-κB and JAK/STAT as a means to attenuate neurodegenerative progression, especially in aging contexts where chronic inflammation becomes self-sustaining.

### 8.2 Limitations and challenges in model translation

Despite the high level of evolutionary conservation, direct translational leaps from *D. melanogaster* to mammalian systems face notable limitations. The absence of an adaptive immune system and the lack of glial diversity particularly astrocytes and microglia mean that *Drosophila* cannot fully recapitulate the cellular complexity of human neuroinflammation (20). While fly glia perform essential roles in synaptic pruning, neurotransmitter clearance, and immune signaling, their functional repertoire remains limited compared to vertebrate glial populations.

Furthermore, significant discrepancies exist in pharmacokinetics and pharmacodynamics between flies and humans. Differences in drug absorption, metabolism, and tissue distribution can lead to substantial variation in therapeutic concentrations and off-target effects, complicating direct extrapolation of dosing regimens and efficacy profiles (51). These limitations underscore the importance of using *Drosophila* primarily for defining causality, prioritizing core molecular targets, and elucidating conserved mechanisms, rather than for predicting clinical outcomes.

Therefore, findings from *Drosophila* models must undergo rigorous validation in mammalian systems, where multilayered regulatory networks, adaptive immunity, and complex tissue interactions can be fully assessed. This tiered approach ensures that insights gained from fly models are translated with appropriate biological context and therapeutic relevance.

### 8.3 Integration with omics, AI/ML-based behavioral analytics, and neuroimmune biomarker discovery

The longitudinal multi-omics datasets generated from *Drosophila melanogaster* neurodegeneration models particularly those expressing human α-Syn have revealed a conserved molecular trajectory characterized by a shift from metabolic maintenance to chronic inflammation, a hallmark of inflammaging. These datasets include transcriptomic, proteomic, and metabolomic layers that capture early dysregulation in mitochondrial function, immune signaling, and stress response pathways, offering a high-confidence molecular signature for tracking disease progression (31).

Integrating these fly-derived signatures with human longitudinal cohort data and peripheral sample analyses (e.g., blood and cerebrospinal fluid proteomics, metabolomics) accelerates the identification of multimodal biomarker panels. These panels are particularly valuable for non-invasive monitoring of the systemic inflammatory component of neurodegeneration, enabling the detection of pre-symptomatic stages driven by peripheral immune dysregulation (54). Such integration bridges experimental models with clinical reality, enhancing translational fidelity.

Moreover, the application of Artificial Intelligence (AI) and Machine Learning (ML) to these complex datasets is critical for refining network modeling, predicting therapeutic efficacy, and repurposing drug targets. AI/ML algorithms can detect subtle behavioral phenotypes in *Drosophila*, such as sleep fragmentation, locomotor decline, and circadian rhythm disruption, which correlate with molecular changes and mirror early symptoms in human neurodegenerative diseases. These tools also enable cross-species data harmonization, facilitating the extrapolation of conserved molecular patterns across fly and human systems (55).

Together, this integrative approach combining *Drosophila* omics, human biomarker validation, and AI-driven analytics represents a powerful pipeline for precision neuroimmune diagnostics and early-stage therapeutic intervention.

## 9 Future perspectives

### 9.1 Need for integrative neuroimmunology using multi-omics and *in vivo* imaging

Future *D. melanogaster* research must embrace truly integrative neuroimmunology to decode the complex, non-linear networks that govern neuroimmune interactions. This requires the convergence of multi-omics platforms including genomics, transcriptomics, proteomics, and metabolomics to map how inflammatory signals disrupt central metabolic pathways, such as amino acid biosynthesis, lipid metabolism, and energy homeostasis, thereby linking neurodegeneration with systemic metabolic disorders (54).

These omics layers, when analyzed longitudinally, reveal dynamic shifts in TOR signaling, immune activation, and neurobehavioral plasticity, especially under environmental stressors. For example, predator-induced memory formation in *Drosophila* is modulated by the gut microbiome–TOR axis, illustrating how peripheral metabolic cues shape central neural outcomes (56). Such findings underscore the need to integrate host–microbiome–immune–neural interactions into neurodegenerative frameworks.

Complementing molecular analysis, high-resolution *in vivo* imaging is essential to capture the real-time kinetics and spatial dynamics of glial–neuronal interactions, synaptic remodeling, and immune cell migration. Recent studies have visualized Draper-mediated glial engulfment and neuropil refinement, offering mechanistic insight into how glia sculpt neural circuits during development and degeneration (19). These imaging tools, when paired with omics data, enable spatiotemporal mapping of neuroimmune events and facilitate predictive modeling of disease trajectories.

Together, the integration of multi-omics, live imaging, and AI/ML-based analytics will empower *Drosophila* research to move beyond reductionist paradigms and toward systems-level neuroimmunology, accelerating biomarker discovery and therapeutic innovation.

### 9.2 Expanding *Drosophila* neuroimmune research to aging, metabolic, and environmental stress paradigms

To enhance translational relevance, *D. melanogaster* neurodegeneration models must evolve to incorporate multi-factorial risk factors that reflect the complexity of human disease. This includes intersecting genetic predisposition with aging, metabolic dysregulation, and environmental stressors, which collectively shape the neuroimmune landscape and influence disease onset and progression. Recent studies have demonstrated that withaferin A, a plant-derived steroidal lactone, exhibits geroprotective effects in aging *Drosophila*, improving lifespan, stress resistance, and locomotor function. These benefits are mediated through modulation of oxidative stress, immune tone, and proteostasis, validating the fly as a platform for screening anti-aging compounds with neuroprotective potential (42).

In parallel, the gut–brain–immune axis has emerged as a critical regulatory node. A landmark study revealed that specific microbiome compositions interact with the host TOR signaling pathway to modulate aversive memory formation in flies exposed to predator cues. This interaction influences neurobehavioral plasticity and links peripheral metabolic signals to central cognitive outcomes, reinforcing the need to integrate microbiome–immune–neural crosstalk into neurodegenerative frameworks (56).

Moreover, dietary interventions such as high-sugar or high-fat diets in *Drosophila* have been shown to induce systemic inflammation, insulin resistance, and accelerated neurodegeneration, mimicking metabolic syndrome–linked cognitive decline in humans. These models allow for the dissection of nutrient–immune–neural interactions, particularly how diet-induced stress alters glial function, synaptic integrity, and immune tone (57).

Environmental stressors such as oxidative agents, temperature fluctuations, and pathogen exposure also modulate neuroimmune outcomes. For instance, chronic exposure to paraquat induces dopaminergic neuron loss and Imd pathway activation, recapitulating Parkinsonian features and validating the fly as a model for environmentally triggered neurodegeneration (58).

Together, these findings underscore the importance of expanding *Drosophila* neuroimmune research to include aging, diet, microbiome variability, and environmental challenges. Such integrative models will enable the identification of context-sensitive therapeutic targets and facilitate the development of precision interventions that account for age, metabolic state, and immune history.

### 9.3 AI-assisted phenotyping and network modeling of immune interactions

The exponential growth of multi-dimensional biological datasets including behavioral phenotypes, transcriptomic profiles, proteomic maps, and metabolomic signatures necessitates the enhanced utilization of AI and ML to extract actionable insights. These computational approaches are particularly valuable in *D. melanogaster* neuroimmune research, where subtle behavioral changes (e.g., sleep fragmentation, locomotor decline, circadian disruption) often precede molecular pathology. AI-assisted models are essential for analyzing complex, nonlinear phenotypes and large-scale omics datasets, enabling the identification of hidden patterns, predictive biomarkers, and causal regulatory nodes (55). For example, supervised learning algorithms have been used to classify neurodegenerative stages based on multi-omics trajectories, while unsupervised clustering reveals novel disease subtypes and immune signatures.

Future applications will leverage Generative Adversarial Networks (GANs) and Transformer-based architectures to perform complex network modeling of inter-organ immune interactions, such as gut–brain–immune crosstalk. These models can simulate the effect of therapeutic pathway manipulations, predict off-target immune responses, and refine drug repurposing strategies with enhanced precision and cost-effectiveness (55, 64). Moreover, bioinformatics pipelines integrating AI/ML can harmonize *Drosophila* datasets with human clinical cohorts, facilitating cross-species biomarker discovery and translational validation. This is particularly relevant for tropical and age-related diseases, where multi-omics integration and AI-driven phenotyping accelerate the identification of context-sensitive therapeutic targets (65).

Together, these computational advances position AI/ML as a cornerstone of next-generation neuroimmune research, transforming *Drosophila* from a reductionist model into a predictive systems biology platform.

## 10 Conclusion

*D. melanogaster* has firmly established itself as an indispensable model system for dissecting the core mechanisms of neuroimmune crosstalk underlying neurodegeneration. Recent findings conclusively demonstrate that NDs such as PD and AD are systemic disorders, where peripheral immune dysregulation originating from the gut–immune axis and transmitted via conserved cytokine signaling pathways like Upd3/IL-6 acts as a causal and early driver of both CNS pathology and peripheral motor dysfunction.

The fly model’s capacity to define causality, prioritize conserved molecular targets, and reveal stage-specific therapeutic limitations has been exemplified in studies evaluating compounds like Curcumin, which show adult life-phase–specific dopaminergic neuroprotection. These effects are mediated by differential regulation of brain-specific molecular pathways, emphasizing the need for temporal precision in therapeutic design.

Moreover, *Drosophila* has illuminated the role of NF-κB signaling, particularly through regulators like Pirk, in modulating brain–immune interactions. Loss of Pirk leads to exacerbated neuroinflammation, reinforcing the importance of innate immune tone in maintaining CNS integrity. The Upd family cytokines like Upd1, Upd2, and Upd3 serve as functional analogs of mammalian IL-6, orchestrating inter-organ immune communication and linking gut-derived signals to neural outcomes.

Recent multi-omics studies have further revealed that inflammaging, driven by chronic immune activation and metabolic repression, precedes overt neurodegeneration and can be tracked using peripheral biomarker panels derived from *Drosophila* and validated in human cohorts. These insights are amplified by AI-assisted phenotyping and network modeling, which enable the prediction of therapeutic efficacy and the repurposing of immune-modulatory compounds with enhanced precision.

As highlighted in recent reviews, the field may be underutilizing the full potential of fruit flies in inflammation-led drug discovery, especially given their scalability, genetic tractability, and ability to model complex neuroimmune interactions under aging, dietary, and environmental stress paradigms. Embracing this potential will be critical for advancing next-generation therapeutics for PD, AD, and related disorders.

By focusing interventions on conserved systemic drivers of inflammation, and integrating multi-omics platforms with AI-assisted network analysis, *Drosophila* research continues to serve as a high-fidelity translational bridge, accelerating the discovery of disease-modifying therapies and supporting precision medicine pipelines for neurodegenerative disorders.
